# Association of *AIRE* Polymorphism and the Susceptibility to Multiple Sclerosis in Iranian Population

**Published:** 2018

**Authors:** Tahereh Sadeghian-Rizi, Fereshteh Alsahebfosoul, Mohammad Kazemi, Hossein Khanahmad, Ali Jahanian-Najafabadi

**Affiliations:** 1. Department of Pharmaceutical Biotechnology, Faculty of Pharmacy, Isfahan University of Medical Sciences, Isfahan, Iran; 2. Department of Immunology, Faculty of Medicine, Isfahan University of Medical Sciences, Isfahan, Iran; 3. Department of Genetics and Molecular Biology, Faculty of Medicine, Isfahan University of Medical Sciences, Isfahan, Iran

**Keywords:** *AIRE*, Iran, Multiple sclerosis, Single-nucleotide polymorphism

## Abstract

**Background:**

Multiple Sclerosis (MS) is the most common cause of neurologic disability in young adults. Recently, the *AIRE* gene was identified as a genetic risk factor for several autoimmune diseases in genome wide association studies. The aim of this study was to further investigate the possible role of the *AIRE* gene in susceptibility to MS in Iranian population.

**Methods:**

A total of 112 MS patients and 94 ethnically matched controls were included in the study. The Single-Nucleotide Polymorphism (SNP) (rs1800520, C>G) with a global MAF=0.2282/1143 was selected and genotyped using HRM real-time PCR method.

**Results:**

Results showed that *AIRE* SNP rs1800520 was significantly less common in the MS patients than in healthy controls (17.8 *vs*. 28.7%, pc=0.032, OR=0.54,95% CI 0.279, 1.042). Also, the frequency of allele G was significantly higher among the control group than in the case group (37.77 *vs*. 25%, pc=0.014). Interestingly, mRNA transcribed on the rs1800520 SNP showed decreased free energy than the wild type suggesting that its increased stability may be responsible for the different activities of the polymorphic AIRE molecule.

**Conclusions:**

This is the first study investigating the relationship between *AIRE* gene and the susceptibility to MS. These results indicated that the rs1800520 SNP is not a susceptibility gene variant for the development of MS in Iranian population.

## Introduction

Multiple Sclerosis (MS) is an autoimmune, common and severe CNS disease that is characterized by demyelination, chronic inflammation, axonal and oligodendrocyte pathology, and progressive neurological dysfunction^1^. Prevalence rates for MS vary between 2 and 160 per 100,000 in different countries, and more than 2 million individuals are affected by this disease worldwide^2^. The incidence and prevalence of MS in Iran has been increasing rapidly, especially in females^3^. Extensive epidemiological data confirm that genetic variation is an important determinant of susceptibility to MS, and suggest that such variation also influences the timing of symptom onset, the course of the disease, and the treatment response^4^. MS as an autoimmune disease is caused by a breakdown in central or peripheral tolerance toward self-antigens, allowing autoreactive pathogenetic T and B cells clones arising, and a complex-mix of genetic and environmental factors is believed to mediate this breakdown^5–7^. The co-occurrence of autoimmune diseases in the same individual has prompted several studies aimed to recognize shared pathophysiological mechanisms.

Among genetic factors, variant of *AIRE* (Autoimmune Regulator) gene has been correlated with autoimmune manifestations in the APECED^8,9^, rheumatoid arthritis^10,11^, the immune deficiency omenn syndrome^12^, alopecia aerate^13^, and lupus like panniculitis in patients with APECED^14^ but not with type I diabetes, addison disease^15^ and Graves’ disease^16^, myasthenia gravis^17^ as well as inflammatory bowel disease^18^. AIRE protein acts as a powerful transcriptional trans-activator. The AIRE protein, mostly localized in the cell nucleus, is composed by specific domains including the amino-terminal HSR domain, the Nuclear Localization Signal (NLS), the Sp100, AIRE1, nucP41/75, DEAF 1 (SAND) domain, two Plan the Homeo-domain (PHD) type zinc fingers, and four LXXLL motifs^19^. The highest level of AIRE expression is in thymus where it is seen in a subpopulation of modularly Thymic Epithelial Cells (mTEC)^4^. In mTECs, AIRE is required for the expression of many Tissue-Restricted self-Antigens (TRAs)^20^. The expression of TRAs in mTECs allows the negative selection of autoreactive lymphocytes. In the absence of functional AIRE, human patients and mice develop multi-organ autoimmune disease due to a defect in thymic negative selection^21^.

On this basis, an attempt was made to analyze SNP rs1800520 in the SAND domain of *AIRE* gene in order to investigate whether this polymorphic genotype could protect or predispose to the development of MS. Among all the *AIRE* SNPs already identified, this SNP was selected following three main criteria concluding: 1) the SAND domain is a conserved sequence motif in nuclear proteins including Sp100 family and plays a key role in transcription regulation, thus mutations at this domain could destabilize the binding of AIRE to TRAs promoter and lower TRAs expression in thymic epithelial cells^22^; 2) the already demonstrated association of this SNP with human autoimmune diseases specially rheumatoid arthritis in which Genome-Wide Association Studies (GWAS) on rheumatoid arthritis and MS show that these diseases share many genetic factors^23^ and 3) the only common frequency (MAF>0.05) SNP leading to a mis-sense mutation (serine to arginine) in AIRE exon and the MAF of this SNP in Asian population is 0.475^17^.

## Materials and Methods

### Study subjects

A total of 112 MS patients (87 women and 25 men) were included in the study. All patients met the MS research center of the Alzahra hospital in Isfahan, Iran. The control population consisted of 94 healthy persons without any autoimmune diseases. Demographic information of patient and control population was presented in supplementary data. Blood samples were obtained from subjects after they provided written informed consent. Genomic DNA was extracted from blood leukocytes using QIAmpDNA Mini Kit (Qiagen, Hilden, Germany) according to the manufacturer’s recommendations and stored at −20°*C*.

### SNP genotyping

The polymorphism rs1800520 (C8723G) was investigated using the allelic discrimination assay by HRM-real time PCR. PCR was performed using two primers flanking the SNP. The assay was conducted with the Rotor-Gene 6000 instrument (Corbett Life Science) and the software Rotor-Gene 6000 series version 1.7 was used to analyze the results. PCR was carried out in three steps: activation, amplification and melting. Primer sequences and HRM-real time PCR conditions were listed in table 1. At least, 10% of all genotyping results were confirmed by sequencing.

**Table 1.** Primer sequences and PCR conditions

**Primer sequences**	
Forward	5′-ATTGCTGACGCCCCTCTT-3′
Reverse	5′-TAGGGCATTACCTGGTGGAG-3′
**PCR conditions**	
Activation	95°*C* for 15 *min*
Amplification	95°*C* for 15 *s*, 60°*C* for 20 *s*,72°*C* for 20 *s*
Melting	ramp from 70°*C* to 95°*C*, raising by 0.2°*C* each step, wait for 2 *s* for each step afterwards

### Prediction of RNA secondary structures of AIRE allelic variants

The potential variations in folding of the RNA secondary structure caused by the SNP rs1800520 of AIRE variants were identified using the RNA structure web servers on the Mathews Lab Web Servers from University of Rochester Medical Center (http://rna.urmc.rochester.edu/).

### Statistical analysis

SPSS (version 18.0, SPSS, Chicago, IL, USA) was used for statistical analysis. Statistically significant differences between genotype frequencies were assessed using univariate analyses such as the Fisher’s exact test for binary variables and the Student t test for continuous variables. The association between the presence of polymorphic genotypes and independent variables was studied using a multivariate logistic regression analysis. The genotype and allelic frequencies were assessed using the Hardy-Weinberg equilibrium (https://www.easycalculation.com/health/hardy-weinberg-equilibrium-calculator.php). Odds Ratio (ORs) and 95% Confidence Intervals (95% CIs) were calculated and p-values lower than 0.05 were considered statistically significant.

## Results

### Decreased frequency of rs1800520 (C8723G) SNP in MS patients compared with controls

Analysis of the polymorphism rs1800520 genotype and allelic frequencies showed that both groups (patients and controls) were in Hardy-Weinberg equilibrium. The genotype and allelic frequencies were re-analyzed by SNPStat software and recessive form was selected as the best model according to the AIC (Table 2). Results demonstrated that *AIRE* SNP rs1800520 was significantly less common in the MS patients than in healthy controls (17.9% vs. 29%, pc = 0.032, OR = 0.54, 95% CI 0.279, 1.04) (Table 3).

**Table 2.** Selection of the best model by SNPstat software

**Model**	**Genotype**	**Case**	**Control**	OR* (95% CI**)	**p-value**	AIC***	BIC****
**Codominant**	C/C	76 (67.9%)	50 (53.8%)	1.00	0.013	199.5	219.4
	C/G	16 (14.3%)	16 (17.2%)	1.03 (0.38–2.76)			
	G/G	20 (17.9%)	27 (29%)	4.03 (1.51–10.79)			
**Dominant**	C/C	76 (67.9%)	50 (53.8%)	1.00	0.048	202.3	218.9
	C/G-G/G	36 (32.1%)	43 (46.2%)	2.11 (1.00–4.48)			
**Recessive**	C/C-C/G	92 (82.1%)	66 (71%)	1.00	0.0031	197.5	214.1
	G/G	20 (17.9%)	27 (29%)	4.01 (1.53–10.53)			
**Overdominant**	C/C-G/G	96 (85.7%)	77 (82.8%)	1.00	0.55	205.9	222.5
	C/G	16 (14.3%)	16 (17.2%)	0.75 (0.29–1.93)			
**Log-additive**	---	---	---	1.84 (1.16–2.93)	0.0079	199.2	215.8

* Odds Ratio,

** Confidence Interval,

*** Akaike Information Criterion,

**** Bayesian Information Criterion.

**Table 3.** Allele and genotype frequency of AIRE rs1800520

	**Case (n=112) %**	**Control (n=94) %**	OR* (95%CI**)	**p-value**
**Genotype frequency**				
CC	67.9%	53.2%		
CG	14.3%	18.1%	0.76 (0.358–1.591)	0.459
GG	17.8%	28.7%	0.54 (0.279–1.042)	0.032
GG+GC	32.1%	47%	0.54 (0.305–0.949)	0.032
**Allele frequency**				
C	75%	62.23%		
G	25%	37.77%	0.562 (0.371–0.851)	0.003

* Odds Ratio,

** Confidence Interval.

### Stratification of the frequency of rs1800520 SNP for age of MS onset and sex

Dependency of the frequency of rs1800520 (C8723G) on independent variables such as sex and age of MS onset was investigated on the patient and control population. For the frequency of rs1055311 (C8385T) SNP, there was no statistically significant difference in males in comparison to females in the patient and control groups and also no significant associations of the tested SNP with sex and age of MS onset were detected in the patient group (Table 4).

**Table 4.** Association of the SNP rs1800520 with the sex and age of MS onset

**Variables**	OR* (95% CI**)	**p-value**
**Sex**	1.542 (0.876–2.715)	0.134
**Sex and age of MS onset**	0.8 (0.314–2.037)	0.640

* Odds Ratio,

** Confidence Interval.

### AIRE polymorphism and mRNA structure

**Figure 1.**
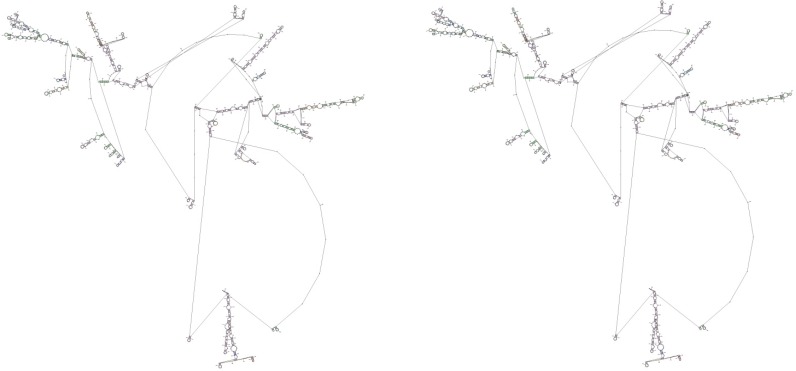
Computerized modeling of AIRE mRNA structures of two haplotype variants in exon 7. A) AGCG allelic variant corresponding to the wild-type genotype; B) AGGG allelic variant corresponding to the C8723G SNP.

In order to characterize the mechanisms determining the different functional activity of AIRE allelic variants at the molecular level, our analysis was focused on mRNA folding structure. Genetic polymorphisms may generate mRNA molecules showing different secondary structures. Interestingly, variants of mRNA secondary structure have been associated with different efficiencies of gene expression depending on the free energy possessed by the single mRNA molecule^18^. Therefore, the mRNA folding structure corresponding to the AIRE allelic non-synonymous variants AGCG, present in the wild-type genotype, and AGGG, present in the rs1800520 (C8723G) SNP, in exon 7 was investigated using the RNA structure web servers on the Mathews Lab Web Servers from University of Rochester Medical Center (http://rna.urmc.rochester.edu/). mRNA transcribed on AIRE AGCG allele showed lower free energy (dG=−1043.5 *kcal/mol*) than the one transcribed on the AGGG allele (dG=−1404.5 *kcal/mol*), suggesting that the latter likely has greater molecular stability than the former (Figures 1A and 1B). In conclusion, mRNA molecules transcribed on the variant AGGG genotype might have a longer half-life and produce more AIRE protein than mRNA molecules transcribed on the wild type AGCG genotype.

## Discussion

AIRE is a transcriptional regulator and is expressed primarily by medullary thymic epithelial cells and cells of the monocytes dendritic cell lineage of the thymus. The expression of AIRE in non-thymic tissues is still controversial^24^. AIRE plays a major role in thymocyte education and negative selection by controlling the expression of peripheral antigens in the thymus^25^. Thus, AIRE is a good functional candidate in autoimmune diseases regardless of the population. In fact, mutations in this gene cause autoimmune polyendocrinopathy candidiasis-ectodermal dystrophy (APECED), which is one of the few known monogenic autoimmune diseases where patients suffer from both endocrine and non-endocrine manifestations. AIRE mutations and polymorphisms have been found in different autoimmune manifestations correlated or not correlated to APECED^26^.

In this research, SNP rs1800520 (C>G) of *AIRE* gene was analyzed in the MS patients in Iranian population. TheS278R replacement by rs1800520 is located in the SAND domain, a conserved sequence motif in nuclear proteins and plays a key role in transcription regulation. *AIRE* SNPs have been positively correlated with some autoimmune diseases and some researchers have investigated SNP rs1800520 with respect to Finnish type 1 diabetes^27^, rheumatoid arthritis^10,11^, melanoma^20^, myasthenia gravis^17^ and APS1^28^. The GWA study of Terao *et al* established the association of SNP rs1800520 with rheumatoid arthritis in the Japanese population but García-Lozano *et al* observed no significant difference in the distribution of the G allele of rs1800520 between rheumatoid arthritis patients and controls in the European population. Conteduca *et al* found that this SNP was significantly more frequent in healthy subjects than in melanoma patients, independently from sex, age and stages of melanoma and concluded that G allele exerts a significant protective effect against melanoma. Zhang *et al* showed that there were no significant differences in frequencies of alleles and genotypes in rs1800520 between myasthenia gravis group and the control group in the Chinese population. Also Turunen *et al* found that this SNP does not seem to contribute to disease susceptibility in Finnish type 1 diabetic patients.

Our study is the first study on the correlation of *AIRE* polymorphism and MS. The increased frequency of rs1800520 (C8723G) SNP in healthy subjects with respect to MS patients suggests that this polymorphism may be associated with an increased expression of AIRE in individuals bearing the corresponding genotypes (GG), thus conferring them a potential protecting against MS development. Our in silico analysis of the folding of the polymorphic AIRE mRNA structures showed that the genotype corresponding to rs1800520 SNP leads to the transcription of a mRNA molecule whose secondary structure is likely more stable than the one of the mRNA coded by the AIRE wild-type genotype. This observation suggests that, in the case ofrs1800520 SNP, AIRE mRNA may have a longer half-life causing increased AIRE protein production and, thereby, leading to higher MS-associated antigen expression in mTECs and negative selection of autoreactive lymphocytes. Further studies are warranted to validate these results and to investigate the underlying molecular mechanisms because the increased frequency of this SNP was shown in many autoimmune diseases. Thus, our results may demonstrate the lack of association between this SNP and MS in Iranian population and may associate MS susceptibility with another *AIRE* SNPs or the same SNP in another population like RA susceptibility association with this SNP in Japanese population but not in European population^10,11^.
